# Immune Checkpoint Inhibitors for the Treatment of Bladder Cancer

**DOI:** 10.3390/cancers13010131

**Published:** 2021-01-03

**Authors:** Antonio Lopez-Beltran, Alessia Cimadamore, Ana Blanca, Francesco Massari, Nuno Vau, Marina Scarpelli, Liang Cheng, Rodolfo Montironi

**Affiliations:** 1 Unit of Anatomic Pathology, Department of Morphological Sciences, Cordoba University Medical School, 14004 Cordoba, Spain; 2 Pathological Anatomy, School of Medicine, United Hospitals, Polytechnic University of the Marche Region, 60126 Ancona, Italy; a.cimadamore@staff.univpm.it (A.C.); m.scarpelli@staff.univpm.it (M.S.); 3 Maimonides Biomedical Research Institute of Cordoba, Department of Urology, University Hospital of Reina Sofia, 14004 Cordoba, Spain; anblape78@hotmail.com; 4 Division of Oncology, IRCCS Azienda Ospedaliero-Universitaria di Bologna, 40138 Bologna, Italy; fmassari79@gmail.com; 5 Medical Oncology, Champalimaud Clinical Center, 1400-038 Lisbon, Portugal; nuno.vau@fundacaochampalimaud.pt; 6 Department of Pathology and Laboratory Medicine, School of Medicine, Indiana University, Indianapolis, IN 46202, USA; liang_cheng@yahoo.com

**Keywords:** bladder cancer, variant histology, urothelium, PD-1, PD-L1, immunotherapy, tumor mutation burden, immune checkpoint inhibitor, biomarker, Durvalumab, Atezolizumab, Nivolumab, Pembrolizumab, Avelumab

## Abstract

**Simple Summary:**

In this review, we examined relevant clinical trial results with immune checkpoint inhibitors in patients with metastatic urothelial cancer. We also focused on the potential of immunotherapy in the adjuvant and neoadjuvant setting or as part of drug combinations. Finally, we briefly review the current landscape of biomarkers of response to immune checkpoint inhibitors, such as programmed death-ligand 1 (PD-L1) expression, tumor mutation burden, molecular subtypes of bladder cancer, and immune-gene expression profiling.

**Abstract:**

A number of immune checkpoint inhibitors (ICIs) have been approved as first-line therapy in case of cisplatin-ineligible patients or as second-line therapy for patients with metastatic urothelial carcinoma (mUC) of the bladder. About 30% of patients with mUC will respond to ICIs immunotherapy. Programmed death-ligand 1 (PD-L1) expression detected by immunohistochemistry seems to predict response to immune checkpoint inhibitors in patients with mUC as supported by the objective response rate (ORR) and overall survival (OS) associated with the response observed in most clinical trials. Pembrolizumab, an anti-PD-1 antibody, demonstrated better OS respective to chemotherapy in a randomized phase 3 study for second-line treatment of mUC. Nivolumab, a PD-1 antibody, also demonstrated an OS benefit when compared to controls. Atezolizumab, Durvalumab, and Avelumab antibodies targeting PD-L1 have also received approval as second-line treatments for mUC with durable response for more than 1 year in selected patients. Atezolizumab and Pembrolizumab also received approval for first-line treatment of patients that are ineligible for cisplatin. A focus on the utility of ICIs in the adjuvant or neoadjuvant setting, or as combination with chemotherapy, is the basis of some ongoing trials. The identification of a clinically useful biomarker, single or in association, to determine the optimal ICIs treatment for patients with mUC is very much needed as emphasized by the current literature. In this review, we examined relevant clinical trial results with ICIs in patients with mUC alone or as part of drug combinations; emphasis is also placed on the adjuvant and neoadjuvant setting. The current landscape of selected biomarkers of response to ICIs including anti-PD-L1 immunohistochemistry is also briefly reviewed.

## 1. Introduction

Bladder cancer is considered one of the most aggressive neoplasms worldwide [1]. Nonetheless, the majority of patients present with the less aggressive non-muscle invasive bladder cancer; and about 30% of patients present with muscle invasive disease, which portend a worse prognosis due to its metastatic potential. The all-stage five-year overall survival (OS) rate for urothelial carcinoma remains about 80%. Typically, advanced disease or relapse after radical cystectomy correlate with the poor outcomes that accompany these patients. Traditionally, first-line therapy of metastatic urothelial carcinoma (mUC) remained unchanged over the last decades and was based on cisplatin combinations as the initial choice [2,3,4]. Unfortunately, nearly all patients will ultimately progress and die from bladder cancer despite the initial response associated with cisplatin-based combinations.

**Figure 1:**
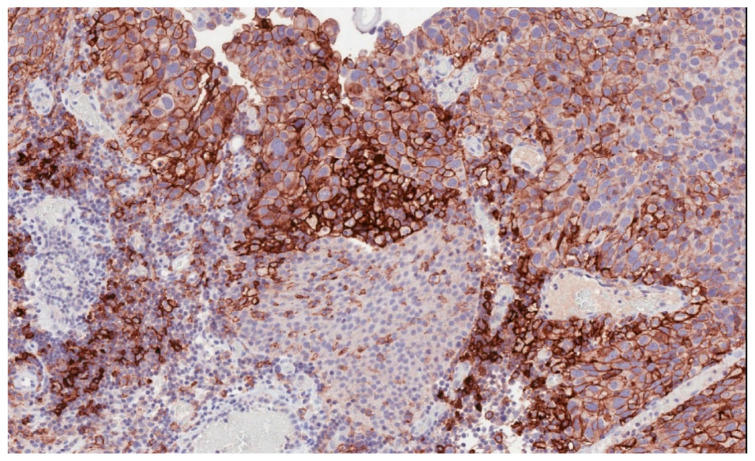
Programmed death-ligand 1 (PD-L1) membranous immunostaining in high-grade urothelial carcinoma (22C3 antibody) (200×).

Immune checkpoint inhibitors have become an increasingly used therapeutic option in many solid tumors [5,6,7,8,9,10]. In bladder cancer, high levels of programmed death-ligand 1 (PD-L1) expression (Figure 1) have been reported to be associated with advanced and aggressive tumors with poor survival outcomes [11,12].

PD-L1 expression by immunohistochemistry seems to be associated with resistance to intravesical bacillus Calmette-Guerin (BCG) therapy [12]. Immune checkpoint inhibitors (ICI) have demonstrated a higher benefit in heavy CD8 immune cell infiltrated tumors and in tumors with high tumor mutational burden (TMB), such as the case of bladder cancer. The mechanism is related to a greater T cell-mediated antitumor immune response elicited by the greater availability of neoantigens, which are able to improve the antitumor immune response [13,14,15].

Atezolizumab was the first PD-L1 inhibitor that received accelerated approval by the Food and Drug Administration (FDA) in May 2016 because of the results derived from a phase 2 trial that demonstrated improved response rates compared to controls [16,17,18]. Thereafter, Nivolumab, Pembrolizumab, Avelumab, and Durvalumab have all shown therapeutic activity in mUC, and, therefore, they have received the FDA approval through different clinical trials reporting important differences in the response to ICI as compared with chemotherapy [19,20,21,22,23,24,25]. However, while Pembrolizumb showed an improved median survival from 7.4 months to 10.3 months (hazard ratio (HR) = 0.73, 95% CI 0.59–0.91; *p* = 0.002) compared to chemotherapy, the Atezolizumab trial did not achieve its primary endpoint, showing no superiority over chemotherapy (median OS of 11.1 months in Atezolizumab arm compared to 10.6 months in the chemotherapy arm (HR = 0.87, 95% CI 0.63–1.21; *p* = 0.41) [22,26]. As an additional difficulty, all agents tested in the trials have associated companion diagnostics tests typically focusing on PD-L1 expression but using different technological platforms, monoclonal antibodies, and cut-off algorithms to determine the level of PD-L1 positivity [27,28,29,30,31,32,33]. Although ICI are effective in metastatic urothelial bladder cancer, just a small proportion of treated patient will find a clear benefit while a high number of patients will be exposed to potentially significant side effects and toxicity with no quality of life or survival improvement. No one single biomarker at this point has been associated with response. Here, we report on the current association between PD-L1 expression, treatment with immune checkpoint inhibitors, and outcomes in patients with mUC of the bladder. The potential value of ICI immunotherapy in the adjuvant or neoadjuvant scenario is also briefly reviewed. Finally, the role of antiPD-L1 immunohistochemistry and other potential predictive biomarkers of immune checkpoint inhibitors are also briefly reviewed.

## 2. Overview on Approved Immune Checkpoint Inhibitors for Metastatic Bladder Cancer

### 2.1. Atezolizumab

Atezolizumab is a humanized anti-PD-L1 IgG1 antibody with minimal binding to Fc receptors. Its use has been approved by FDA on the basis of the results of the IMvigor 210 study [16]. Cohort 2 of this trial included patients who had disease progression during or following platinum-based chemotherapy or within 12 months of neoadjuvant or adjuvant therapy. PD-L1 expression was evaluated on immune cells using SP142 monoclonal antibody in a Ventana platform and a cut-off of 5%. Overall, the reported objective response rate (ORR) after Atezolizumab was 14.8% (CI 11.1–19.3) (46 patients). The reported ORR in patients with low PD-L1 immune cell expression was 9.5% (eight patients) compared to 26% (26 patients) in patients with high PD-L1 immune cell expression. The median OS in those patients receiving second line Atezolizumab was 7.9 months (CI 6.7–9.3 months). At a median follow-up of 11.7 months, sustained responses were seen in 38 out of 45 responding patients (84%), which supports prolonged benefit at least in a proportion of patients. Cohort 1 included cisplatin-ineligible patients who were treated with first-line Atezolizumab at the same dose scheme to cohort 2 [17]. Renal impairment that prevented cisplatin treatment was seen in 70% of patients in cohort 1. An ORR of 23% was reported for cohort 1, in contrast to an ORR of 10% in the control group. A median OS for the entire cohort of 15.9 months was observed, with 21% of the patients receiving therapy for more than 1 year. Contrarily to cohort 2, ORR in cohort 1 appeared to be independent of PD-L1 status (ORR of 28% vs. 21% for high and low PD-L1 immune cells expression, respectively). Median OS was also independent of PD-L1 status (12.3 vs. 19.1 months for high and low PD-L1 immune cells expression, respectively). In both cohorts, the most common adverse events (AEs) were diarrhea, fatigue, and/or pruritus with rare examples of autoimmune phenomena commonly associated with PD-L1 inhibitors including pneumonia, transaminitis, and hypothyroidism.

### 2.2. Pembrolizumab

It is an IgG4 anti-PD1 humanized antibody that binds to programmed cell death protein 1 (PD-1) and blocks the binding of PD-1 with its ligands PD-L1 and programmed death-ligand 2 (PD-L2). Pembrolizumab is an FDA-approved ICI based on a randomized, phase 3 trial [26] known as Keynote-045, which is an open label study that assigned 542 randomly selected patients who had recurred or progressed under platinum therapy. The median OS in the Pembrolizumab arm was 10.3 months compared to the 7.4 months of the chemotherapy group (*p* = 0.002). Akin to the results of other phase 3 studies of PD-1 inhibitors, Progression-Free Survival (PFS) was not increased for Pembrolizumab when compared to chemotherapy, but the objective response rate (ORR) for the Pembrolizumab group was higher than the chemotherapy group (21.1% vs. 11.4%, *p* = 0.001). The observed ORR was comparable between PD-L1 low versus PD-L1 high expression subgroups. PD-L1 expression was evaluated on both tumor and immune cells using the 22C3 monoclonal antibody (Dako assay) and the combined proportion score (CPS). The median OS in the PD-L1 high CPS cohort (CPS > 10) was 8.0 months (CI 5.0–12.3) with Pembrolizumab in contrast to 5.2 months (CI 4.0–7.4) in the chemotherapy cohort. Grade 3 or 4 adverse events were less common in the Pembrolizumab group (15%) as compared to 49.4% in the chemotherapy-treated arm. Pruritus, fatigue, nausea, or diarrhea were the most reported AEs. Pembrolizumab was also approved as first-line therapy in cisplatin ineligible patients in mUC on the grounds of early data from the phase 2 Keynote-052 study [22].

### 2.3. Durvalumab

Granted in May 2017 based on a single-arm phase 1/2 trial including 61 platinum-treated patients with advanced urothelial carcinoma, Durvalumab is an FcR binding deficient anti-PD-L1 antibody [24,34]. The trial enrolled patients that had disease relapse within 1 year of neoadjuvant chemotherapy. The entire cohort had a 31.0% overall response rate; nonetheless, a 46.4% response rate for patients with PD-L1 expressing tumor cells was observed in contrast to 22% for PD-L1 negative carcinomas. The Ventana SP263 assay was performed to evaluate PDL1 status by immunohistochemistry. The FDA approved Durvalumab along with the Ventana SP263 as a companion diagnostic test. Such assay allows identifying patients for Durvalumab using a composite biomarker and a cut-off of 25%. Practically, patients were considered PD-L1 positive if either tumor cells or immune cells showed ≥25% staining by immunohistochemistry, and they were considered negative if both tumor cells and immune cells expressed ≤25% PD-L1.

A recent follow-up study on 191 patients treated with Durvalumab reported an ORR of 17.8% (overall series based on 191 patients showing TC or IC range from low/negative (≤25%) to high (≥25%) that rose to 27.6% in high PD-L1 expressing patients (TC or IC ≥ 25%) [34,35].

### 2.4. Nivolumab

Nivolumab is a fully humanized IgG4 anti-PD1 antibody approved in 2017 as second-line treatment of platinum refractory mUC based on information from the Checkmate 275 trial. This phase 2 study single-arm enrolled 270 patients to receive Nivolumab (3 mg/kg every 2 weeks) [19,20]. PD-L1 expression was assessed through tumor cells stained with 28-8 antibody (Dako PD-L1 IHC kit, Dako North America, Carpinteria, CA, USA). The ORR was about 20% for Nivolumab in contrast to 10% of the control arm. Tumor cell expression of PD-L1 did not correlate the response to Nivolumab (ORR of 28.4%, 23.8%, and 16.1% were noted for tumor cell PD-L1 expression of >5%, >1%, or <1% respectively). Nevertheless, the reported median OS was greater in PD-L1 positive patients compared to patients whose tumor cells expressed ≤1% PD-L1 (11.30 months vs. 5.95 months).

Eighteen percent (48 of 270 patients) experienced grade 3 or 4 adverse events with grade 3 or 4 diarrhea being the most frequent seen after Nivolumab. There were three on-study treatment related deaths: one case each of acute respiratory failure, pneumonitis, and cardiac compromise.

### 2.5. Avelumab

The activity of Avelumab in platinum refractory metastatic bladder cancer has been explored in the single-arm phase 1b JAVELIN clinical trial [36]. Avelumab is an IgG1-type anti-PD-L1 antibody that blocks the link between PD-1 and its ligand PD-L1 but not between PD-1 and PD-L2. A median OS of 13.7 months together with an ORR of 18.2% was initially reported by the JAVELIN trial. Unfortunately, all 44 participating patients developed an adverse event, which included developing infusion reactions in 20% of patients. Nonetheless, there was a trend toward increased survival after 12 weeks of treatment (primary end-point) in patients with high PD-L1 expressing tumors as compared to patients with low PD-L1 expressing tumors (ORR of 53.8% vs. 9.0%, respectively). The JAVELIN trial used the 73-10 monoclonal antibody on a DAKO platform for immunohistochemistry and a cut-off of 5% positive cells to define a case as positive (Dako North America, Carpenteria, CA, USA) [25]. The FDA granted Avelumab for 2nd-line treatment of locally advanced or mUCin platinum-refractory patients. Adverse events noted in more than 10% of patients after Avelumab included infusion reactions (22.8%) and fatigue (12.0%). Importantly, 11.6% of patients experienced an autoimmune adverse event and one treatment-related death due to pneumonitis.

In 2020, Avelumab received the FDA approval for the treatment of patients with locally advanced or metastatic bladder cancer that has not progressed with first-line platinum-containing chemotherapy [37]. The GCISAVE trial (NCT03324282) will assess the effectiveness of Avelumab in combination with gemcitabine/cisplatin in the first-line treatment of locally advanced metastatic bladder cancer. Avelumab is also currently being evaluated in patients with non-muscle invasive UC in combination with BCG (NCT03892642). In patients with advanced UC, combinations of Avelumab with radiation (NCT03747419) and KHK2455 (an indoleamine 2,3-dioxygenase inhibitor; NCT03915405) are also being tested [38]. (Table 1).

## 3. Immune Checkpoint Inhibitors for Bladder Cancer: The Adjuvant Setting

Adjuvant ICI-based therapy has become an extended clinical practice in high-risk patients, in some countries, in particular in patients that have not received neoadjuvant chemotherapy. This practice is mostly derived from melanoma patients in which adjuvant Pembrolizumab has demonstrated better 1-year recurrence-free survival (75.4% vs. 61.0%; HR = 0.57; 98.4% CI, 0.43 to 0.74; *p* < 0.001) in a randomized phase III study [41,42,43]. The rationale behind using this scheme is related to the hypothesis that adjuvant ICI could also work in other high immunogenic tumors such as bladder cancer [42,44]. Currently, several trials are ongoing, both after surgery and after bladder-sparing approaches with chemoradiotherapy to explore the potential survival benefit of adjuvant ICI (Table 2).

## 4. Immune Checkpoint Inhibitors for Bladder Cancer: The Neoadjuvant Setting

Clinical studies to explore ICI applicability in the neoadjuvant setting are currently ongoing, with at least two of them that have reported their results [45]. Atezolizumab was administered in two cycles before surgery in a single arm, phase 2 neoadjuvant trial known as ABACUS, with a ratio defined as of pathologic complete response equal or greater than 20% as end-point. In this trial, 69 patients were recruited; 62 of them had cystectomy following neoadjuvant chemotherapy (NAC) treatment. The reported complete response within this trial was 29%. Twelve percent of the patients had clinically considered severe adverse events; a possible treatment-related patient’s death was also reported [46].

The PURE clinical trial was dedicated to Pembrolizumab with the administration of three cycles in 50 patients after Transurethral Resection Bladder Tumor (TURBT) but before radical cystectomy. Clinical stage T2-T4a N0 (assessed with CT, MRI, or PET/CT) was the most important inclusion criteria together with predominant urothelial histology, residual disease after TURBT, and good general conditions (ECOG PS 0–1). Pathologic complete response (pT0) at the time of surgery was the primary objective (end-point). After pathologic assessment, 42% (21 patients) of them resulted in pT0 after radical cystectomy. Necchi et al. [47] concluded that Pembrolizumab administered in the neoadjuvant setting was safe in patients with muscle invasive bladder cancer (MIBC) and that Pembrolizumab could be a valuable neoadjuvant therapy for MIBC limited to patients with PD-L1–positive or high TMB tumors. The PURE trial reported three patients with grade 3 adverse events with only one patient that had to interrupt therapy with Pembrolizumab. A number of the actively recruiting neoadjuvant trials explore the potential to combine immune checkpoint inhibitors with standard chemotherapy, but currently, there are limited available data (Table 2).

## 5. Is There Any Role of Combination Immunotherapy in Bladder Cancer?

Ongoing trials are designed to explore novel combinations of drugs, for example anti-PD-1/PD-L1 therapy in combination with more classic drugs, thus including intravesical BCG or chemotherapy [48,49]. In this line, combinations of Nivolumab, Ipilimumab, and Cabozantinib have been found safe to treat different genitourinary malignancies [50]; importantly, ICI blockade in BCG-refractory non-muscle invasive bladder cancer is the topic of some ongoing trials, thus opening a new way to treat non-muscle invasive bladder cancer (NMIBC) with aggressive features [51]. In fact, trials evaluating Pembrolizumab [49] (NCT02324582, NCT02808143) or Atezolizumab [52] (NCT02792192) in combination with BCG are still recruiting patients.

Ongoing trials are investigating the combination of ICI with chemotherapy. The rationale behind these trials is the fact that chemotherapy induces immunogenic cell death with a concomitant release of tumor antigens and increases in MHC-I mediated tumor antigen presentation. This may enhance the effects of the immune system within the tumor. Another mechanism is by direct modulation of the quantity and/or activity of immunosuppressive cellular subsets [53,54].

IMvigor-130 is a phase 3 double blind, three-arm, multicenter trial of Atezolizumab as monotherapy or in combination with platinum-based chemotherapy compared to chemotherapy plus placebo in untreated bladder carcinoma patients with locally advanced or metastatic disease [39]. A similar first-line, phase 3, three-arm, multicenter clinical trial was set to investigate Pembrolizumab (KEYNOTE-36) in monotherapy or in combination with platinum-based chemotherapy against standard chemotherapy plus placebo. In addition to this, the issue of chemotherapy as a bio-modulator of response after immune checkpoint inhibitors has been addressed by two recent publications. Gomez de Liaño et al. [55] analyzed the results on the response of 270 patients with urothelial carcinoma and progressive disease (PD) treated with ICI (69 frontline, 201 later line). Of the patients, 57% of frontline-ICI-PD and 34% of later-line-ICI-PD patients received subsequent systemic therapy, which eventually had an impact on overall survival as demonstrated by multivariate analysis (frontline: HR 0.22, 95% CI 0.10–0.51, *p* < 0.001; later line: HR 0.22, 95% CI 0.13–0.36, *p* < 0.001). In the group of patients who progressed after frontline ICI, there was a respective median OS with and without standard systemic therapy (SST), of 6.8 months (95% CI 5.0–8.6) or 1.9 months (95% CI 0.9–3.0). In this particular study, high disease burden was defined as metastases in three or more different anatomic sites predicted worse survival (HR 2.49, *p* = 0.03; simultaneous liver/bone metastases: HR 3.93, *p* = 0.03). In the group of progressing disease after later-line ICI, predictors of survival included the response to ICIs (HR 0.37, *p* = 0.03), longer exposition to ICIs (HR 0.89, *p* = 0.002), and/or bone metastasis (HR 2.42, *p* < 0.001). Therefore, high disease burden might be a clinical factor predictive of first-line immunotherapy failure [55]. The potential benefit of chemotherapy as a treatment in patients following a PD-1 inhibitor as compared with chemotherapy alone was recently investigated by Kato et al. [56]. The study included 243 patients in the chemotherapy after PD-1 cohort and 1196 controls. The reported ORR was 18.9% for the patients treated by chemotherapy following ICI and 11.0% for the control cohort (ORR ratio 1.71; 95% CI 1.19 to 2.46; *p* = 0.004). The authors concluded that a synergistic antitumor effect could be seen when chemotherapy is administered to patients that have received previous PD-1 inhibitors, and that the synergistic effect seems to be transitory and therefore of limited clinical value. Similar synergistic observations come from radiotherapy in line with the potential of radiotherapy to become a bio-modulator to induce PD-L1 expression in some tumors; this field would benefit from further research [57,58].

In addition, combination therapy using both anti-PD1 along with anti-Cytotoxic T-Lymphocyte Antigen 4 (CTLA4) therapeutics seems mechanistically adequate. CTLA-4 is a protein receptor expressed on activate T-cells that binds B7-1 and B7-2 on antigen-presenting cells [59]. The signaling activated by both receptors, CTLA-4 and PD-1, leads to the inhibition of AKT: CTLA-4 via the protein phosphatase PP2A preserving the activation of the phosphoinositide 3-kinase (PI3K) pathway and PD-1 via the PI3K pathway. AKT regulates the production of IL-2, which is a key regulator of the activity and survival of lymphocytes [60]. The Phase 1/2 CheckMate-032 trial explored the safety and efficacy of the combination Ipilimumab and Nivolumab versus Nivolumab alone in different advanced or metastatic solid tumors, including a cohort of patients with advanced or metastatic bladder cancer. The highest response rate (38%) was achieved in the combination arm (Nivolumab 1 mg/kg plus Ipilimumab 3 mg/Kg) compared to the 25.6% and 26.9% of the Nivolumab alone (3 mg/kg) and Nivolumab 3 mg/kg plus Ipilimumab 1 mg/kg arms, respectively. The response rate rose to 58% when only PD-L1 positive patients were considered. The median OS of this group was 15.3 months (95% CI, 10.1–27.6), and 9.9 months in the Nivolumab alone (3 mg/Kg) arm (95% CI, 7.3–21.1). However, grade 3–4 adverse events were more frequent in the combination group compared with the Nivolumab monotherapy arm (39% of grade 3–4 AEs vs. 27%, respectively) [61]. The randomized, multicenter, CheckMate-901 phase 3 clinical trial explores the combination of Nivolumab plus Ipilimumab in the first-line setting against the combination of Nivolumab plus standard chemotherapy or chemotherapy alone in previously untreated unresectable or metastatic urothelial cancer. The trial aimed to enroll 897 patients, and it still recruiting. Results of the randomized phase 3 DANUBE trial (NCT02516241) have been recently published. The trial investigated the OS in patients who received Durvalumab (PD-L1 inhibitor), with or without tremelimumab (CTLA-4 inhibitor), compared to standard of care chemotherapy as a first-line treatment for metastatic urothelial carcinoma. The study did not meet the co-primary end-points, since Durvalumab alone and the combination therapy did not show a significant advantage in terms of OS compared to standard chemotherapy in the PD-L1 positive patients and in the intention-to-treat population, respectively [40]. A phase 2 study also investigated the combination of gemcitabine and cisplatin plus Ipilimumab vs. chemotherapy alone in patients with metastatic urothelial carcinoma. The objective response rate was 69% with 17% of patients achieving a complete response. However, chemotherapy + Ipilimumab did not achieve the primary end-point [62].

Other targets for immunotherapy being explored include CD73, an immune-modulator recently identified as potential target that is part of an ongoing phase 1b clinical trial testing the combination of Pembrolizumab and anti-CD73 in a variety of malignancies, including bladder urothelial carcinomas [63].

A recent interesting observation is related to improved OS with anti-CTLA-4 in males vs. females (HR 0.65, 95% CI 0.55–0.77 vs. HR 0.79, 95% CI 0.65–0.96, *p=* 0.078). However, the results observed with anti-PD-1were not statistically significant neither for OS (males vs. females) nor for PFS (males vs. females) [64].

## 6. Biomarkers for PD-1/PD-L1 Blockade in Bladder Cancer

### 6.1. PD-L1 Expression

PD-L1 expression detected by immunohistochemistry is seen in about 20 to 30% of urothelial carcinomas of the bladder [65]. Reportedly, high levels of PD-L1 expression assessed by immunohistochemistry may in fact indicate more aggressive bladder tumors as seen by its association with increased pathologic stage at resection and increased all-cause mortality, this in fact indicates that PD-L1 expression is prognostic in terms of outcome [11]. Therefore, this scenario needs to be considered in assessing the role of PD-1/PD-L1 as a predictor of ICI therapy. In bladder cancer, the reported variability of results using PD-L1 staining assays as a single biomarker across different clinical trials highlights the difficulties in our clinical practice when relying on a single marker. The range of results is widely variable and shows a strong association with overall response, as is the case with Durvalumab using a PD-L1 biomarker evaluated using the required composite analysis for patient selection [24] (Table 3) to no association, as noticed with Atezolizumab as second-line therapy in an IMvigor Cohort 2 trial [16], in Keynote-045 (Pembrolizumab [26]), and in Checkmate-275 (Nivolumab [20]). The reason behind the reported discrepancies seems to be related to the use of four available assays for PD-L1 scoring using immunohistochemistry, each of those with their own interpretative algorithm and with different technological platforms for assessment. For example, Dako immunohistochemical assay with the 22C3 and 28-8 antibody clones is in use for clinical trials of Pembrolizumab and Nivolumab, respectively. Nonetheless, Durvalumab and Atezolizumab used SP263 and SP142 antibody clones, respectively, and the Ventana immunohistochemical platform assay [30,66,67].

Reportedly, the immunohistochemistry of SP142 assay showed significantly fewer PD-L1 positive tumor cells; meanwhile, PD-L1 assessed on tumor cells was comparable between the 22C3, 28-8, and SP263 assays [68,69]. Consequently, it seems unlikely that PD-L1 as a single biomarker will effectively guide treatment decisions due to limitations with its positive or negative predictive value.

### 6.2. Molecular Subtype of Bladder Cancer

The molecular subtype classification of urothelial cancer based on the recent development of the so-called “Cancer Genome Atlas” (TCGA) has recently been assessed in several trials as a predictor of the response to PD-1/PD-L1 mediated immunotherapy [70]. For example, the IMvigor210 study cohort 2 (post-chemotherapy) classified the cohort of patients into luminal (*n* = 73) or basal (*n* = 122) molecular subtypes according to TCGA. PD-L1-positive immune cells enrichment was a characteristic of the basal subtype (60% vs. 23%), as was the expression in of PD-L1 in tumor cells (39% vs. 4%) [70,71].

Response to treatment with Atezolizumab was present in all TCGA molecular subtypes, but a higher response rate was noted in the subtype defined by the luminal cluster 2 (*p* = 0.0017, ORR = 34%) compared to the other clusters, luminal cluster 1, basal cluster 1, and basal cluster 2 (ORR 10%, 16%, and 20% respectively). The subsequent analysis of cohort 1 of IMvigor reported the highest response rate in the luminal cluster 2 group (*n* = 11/37, seven partial responses and four complete responses) after treatment with Atezolizumab [38].

Following the same rationale, TCGA related molecular subtypes were also explored in the Checkmate-275 phase 2 trial of Nivolumab; conversely, basal 1 subtype tumors had the highest rate of response in this study (7/23, ORR 30%), followed by the luminal cluster 2 tumors treated with Nivolumab that showed about 25% ORR. Pre-analytics issues including the quality of tissue preservation, fixation, and sample sources are the suggested reasons to explain the reported discrepancies in the metastatic bladder cancer molecular subtypes.

### 6.3. Tumor Mutational Burden

Reportedly, a durable response to ICI in metastatic bladder cancer is connected to the mutational load or tumor mutation burden (TMB) present in a given tumor as well as the number of related neoantigens [13,16]. Available data indicate that TMB is in fact a more robust biomarker than others, including PD-L1 immunohistochemistry, the presence of TILs (tumor-infiltrating lymphocytes), or some clinico-pathologic variables [13,16].

Tumor-related neoantigens have been traditionally identified by exome sequencing and may be validated using T-cell activation methods. Available data indicate that there have been few shared neoantigens, and most identified neoantigens seems to be specific to a given patient; consequently, high non-synonymous TMB is seen typically related to an increased number of neoantigens; this would initially explain the reported data from exome sequencing showing a correlation between TMB and the positive response to immune checkpoint inhibitors immunotherapy.

A subgroup of IMvigor210 Cohort 2 in which 315 cancer-related genes were analyzed showed a higher tumor mutation load in patients who responded as compared to non-responders (*p* < 0.0001; 12.4 per megabase vs. 6.4 per megabase) [16]. However, other related analyses of a subset of 150 patients from IMvigor Cohort 2 did not show a positive correlation between TMB, molecular taxonomic subtype, or the smoking status of the patients, thus suggesting that TMB may better predict response to PD-L1-related ICI in urothelial cancer independently of these factors. On the other side, data from 119 samples in cohort 1 of IMvigor 210, in which TMB was determined, resulted in a positive correlation toward better OS in the highest quartile of TMB (>16 to <62.2 mutations per megabase) compared to quartiles 1 to 3 [17].

In addition, the fact that patients with higher TMB favorably responded to Nivolumab and patients with low or medium TMB values receiving Nivolumab had lower progression-free survival compared to patients receiving chemotherapy alone support a role of TMB as a predictor of ICI mediated therapy. In practical terms, patients having a combination of two biomarkers including high PD-L1 and high TMB might experience durable response following ICI therapy. Some data suggest that a combined TMB and PD-L1 predictive signature might start as earlier as PD-L1 positive above 1% and TMB above the median. However, uncertainties on the role of TMB in ICI therapy remain due to limited observations suggesting that some patients with lower values of TMB may in fact respond to ICI-related immunotherapy [51,72]. Reportedly, the large variability in applied criteria to define TMB in different studies challenges the clinical utility of TMB as a predictive biomarker for ICI-related immunotherapy.

### 6.4. Immune-Gene Expression Profiling

A clinically important limitation when using PD-L1 status as a biomarker in the process of predicting the response to immune checkpoint inhibitors is related to the fact that it provides information on the tumor microenvironment based on a single parameter only to segregate the so-called “hot” from “cold” tumors [60]. RNA-based immune-gene expression profiling has the advantage of providing and quantifying data from multiple tumor cells in a given sample, thus providing more fully representative information of the tumor microenvironment. Therefore, immune-gene expression profiling identifies more accurately the inflammatory status of a tumor by quantifying mRNAs to indirectly assess the status of cell surface proteins, cytokines, and chemokines that define “hot” tumors better than using only the expression of PD-L1 by immunohistochemistry [38].

One study investigated an interferon-gamma (IFN-γ) related signature including 25-IFN-γ related genes in 177 specimens of metastatic bladder cancer assess on biopsies prior to ICI treatment in the Checkmate 275 study with Nivolumab. Higher versus lower values in the IFN-γ gene signature score correlated well with response to Nivolumab (*p* = 0.0003, 20/59 patients with high IFN-γ signature with complete or partial response relative to similar parameters in only 19/118 patients showing medium or low IFN-γ expression signature) [73].

Similar to what has been observed in TMB-related studies, the observed negative predictive value of the immune-gene-related panel remains problematic and difficult to explain, due to the fact that some positive responses were noticed in patients with a non-inflamed cytokine signature; therefore, more research is needed in this area. Tang et al. have recently investigated the gene expression profiles of 29 immune gene sets in three independent databases. Based on single-sample Gene Set Enrichment Analysis (ssGSEA) scores for 29 immune gene sets, they were able to define four immuno-subtypes of bladder cancer (referred to as C1–C4) [74]. C2 is an immune-infiltrating type showing the highest ssGSEA score and the highest degree of immune infiltration, while C4 is an immune “desert” type with the lowest immune score. Moreover, the C2 subtype has the best OS, recurrence free-survival, and PFS, whereas C4 has the worse OS. Such subtypes also differ in sensitivity to immunotherapy and chemotherapy. Prediction models estimated that C2 was most sensitive to anti-PD-1 therapies but also conventional chemotherapy with gemcitabine and bleomycin. On the other hand, cisplatin and doxorubicin might be more effective in the C4 subtype [74].

### 6.5. Other Potential Biomarkers

Microsatellite instability (MSI) has been associated with higher sensitivity to ICI regardless of the histotype and organ of origin. This led to the approval of Pembrolizumab for first tissue/site agnostic indication [75].

Tumors with MSI and with DNA damage repair deleterious mutations have a higher load of insertions/deletions that make these tumors more sensitive to ICI. Indeed, it was strongly associated with response to anti PD-1 and anti PD-L1 agents and survival outcome in patients with mUC treated with Nivolumab and Atezolizumab [76,77].

Tumor-infiltrating lymphocytes (TILs) in urothelial carcinoma were correlated with response, improved overall survival, and disease-free survival [78].

According to Vidotto et al., the presence of a basal subtype, CD8^+^ high TILs, and a high expression of *PD-1, LAG-3, IDO1, CTLA-4*, and *PD-L1* was associated with better prognosis and decreased disease recurrence [79]. On the other hand, tumors with a higher expression of *TGFβ* and its receptors and lacking CD8^+^ TILs were non-responsive to Atezolizumab, thus supporting the hypothesis that high TGFβ expression leads to immune exclusion [80].

## 7. Conclusions

A major paradigm shift in bladder cancer medicine was related to the FDA approval and launch of Avelumab, Pembrolizumab, Durvalumab, Atezolizumab, and Nivolumab to treat patients with mUC being previously treated with chemotherapy.

The use of drug combinations of Anti-PD-L1/PD-1 and Anti-CTL4 seems to be particularly important in metastatic bladder cancer and is now part of several ongoing clinical trials.

High disease burden is defined as a high number of metastatic sites involvement and specific patterns of disease progression, which are clinically accessible parameters that are predictive of first-line ICI based immunotherapy failure. That is, three or more metastatic sites or simultaneous liver/bone metastases may predict worse overall survival. Meanwhile, longer exposure to immune checkpoint inhibitors and bone metastasis only may be predictive of better survival.

Standardized and reproducible biomarkers are also important needs in the clinical scenario of selecting the right therapeutic option; in fact, available individual biomarkers revealed not enough power and reproducibility to predict the response to ICI-based immunotherapy in a given patient. The current clinical scenario would most probably benefit from composite forms of generated data including the PD-L1 immunohistochemistry together with tumor mutation burden and immune-gene expression profiling with special reference to T-cell gene signatures. Of note is the observation made in the clinical trial Checkmate 026 that certain patients whose tumors show low TMB may better respond to systemic chemotherapy, which may benefit from further research.

Reportedly, several types of urologic and non-urologic tumors showing mismatch repair (MMR) defects may be responsive to Pembrolizumab, and this is independent of their tissue of origin; then, assessing MMR status in bladder malignancies might have a role in certain cases. Unfortunately, the low number of bladder cancer with such alterations precludes a limitation in practice.

A different scenario of potential clinical interest might be to concentrate ICI associated research on response biomarkers not only in positive predictive ones but also on negative biomarkers of ICIs response or predictive of immune-related adverse events. Clinically challenging situations occasionally seen in bladder cancer patients such as hyperprogression and pseudoprogression deserve research attention [81,82]. Novel potential lines of research might include the application of artificial intelligence to integrate clinical information with molecular data (big data analysis) that might contribute to this new field of research by uncovering clinically relevant biomarkers that are predictive of response or no response, or maybe, predictive of immune-related adverse events. Naturally, much research is yet needed; nonetheless, a combination of classic clinico-pathologic parameters with information-technology generated data together with genomic profiling might be the future of personalized therapy for bladder cancer.

## Figures and Tables

**Table 1:** Overview of immune checkpoint trials in bladder cancer.

Trial (Year)	Phase	Treatment Arms	*N*	Disease Stage	Line of Therapy	Characteristics
IMvigor210 (2016) [16]	2	Atezolizumab monotherapy in c-DDP unfit	123	mMIBC	1st	ORR: 23% (9% cRR) mPFS: 2.7 mo mOS: 15.9 mo
IMvigor130 (2020) [39]	3	Atezolizumab + platinum-based chemo (A) vs. Atezolizumab monotherapy (B) vs. platinum-based chemo (C)	1213	mMIBC	1st	mPFS: 8.2 vs. 6.3 mo (A vs. C) (*p* = 0.007) mOS: 16 vs. 13.4 mo (A vs C) (*p* = 0.027) mOS: 15.7 vs. 13.1 mo (B vs. C)
JAVELIN Bladder 100 (2020) [37]	3	Avelumab (A) maintenance + BSC vs. BSC	700	mMIBC	1st	mOS: 21.4 vs. 14.3 mo (A vs. BSC, all pts) (*p* = 0.001) mOS: NE vs. 17.1 mo (A vs. BSC, PD-L1 ≥ 5% TC+) (*p* < 0.001)
DANUBE (2020) [40]	3	Durvalumab (D) monotherapy vs. D + Tremelimumab (T) vs. platinum-based chemo	1032	mMIBC	1st	mOS: 14.4 vs. 12.1 mo (D vs. chemo, PD-L1 +) (*p* = 0.30) mOS: 15.1 vs. 12.1 (D + T vs. chemo, all pts) (*p* = 0.075)
CheckMate275 (2017) [20]	2	Nivolumab after platinum-based chemo	270	mMIBC	2nd	ORR: 19.6% (52/265). Paters of responses unrelated to PD-L1 status
KEYNOTE-045 (2017) [26]	3	Pembrolizumab (P) vs. chemo-regimen (TXT or PTX or vinflunine)	542	mMIBC	2nd	mOS: 10.3 vs. 7.4 (P vs chemo, all pts) (*p* = 0.002) mOS: 8.0 vs. 5.2 (P vs chemo, PD-L1 status CPS ≥ 10%) (*p* = 0.005)

**Abbreviations**: N, number of patients; NMIBC, non-muscle invasive bladder cancer; BCG, bacillus Calmette-Guerin; cRR, complete response rate; MIBC, muscle-invasive bladder cancer; pCR, pathologic complete remission; pts, patients; PD-L1, programmed cell-death ligand 1; mMIBC, metastatic muscle-invasive bladder cancer; c-DDP unfit, cisplatin-ineligible; I, first-line; ORR, objective response rate; mPFS, median progression-free survival; mo, months; mOS, median overall survival; mo, months; chemo., chemotherapy; BSC, best supportive care; NE, not estimable; TXT, docetaxel; PTX, paclitaxel; II, second-line; CPS, combined positive score.

**Table 2:** Adjuvant and neoadjuvant immunotherapy trials in bladder cancer.

Trial Identifier	Phase	*N*	Line of Therapy	Characteristics
NCT02632409	3	640	After surgery and/or neoadjuvant chemotherapy	Adjuvant Nivolumab (CheckMate 274)
NCT02450331	3	700	After surgery and/or neoadjuvant chemotherapy	Adjuvant Atezolizumab (IMvigor 010/WO29636)
NCT02736266	2	90	Neoadjuvant prior to chemoradiation	Neoadjuvant Pembrolizumab for muscle invasive bladder cancer (PURE-01)
NCT02365766	1/2	81	Neoadjuvant	Neoadjuvant Pembrolizumab + gemcitabine vs Pembrolizumab + gemcitabine/cisplatin
NCT02845323	2	44	Neoadjuvant	Neoadjuvant Nivolumab + urelumab vs Nivolumab monotherapy
NCT02690558	2	39	Neoadjuvant	Neoadjuvant Pembrolizumab + gemcitabine/cisplatin
NCT02662309	2	85	Neoadjuvant	Neoadjuvant Atezolizumab (ABACUS)

**Table 3:** Commonly used anti PD1-PDL1 antibodies to guide immune checkpoint blockade immunotherapy in bladder cancer.

Properties	Avelumab	Durvalumab	Atezolizumab		Nivolumab		Pembrolizumab	
PD-L1 assay (antibody)	Dako 73-10	Ventana SP263	Ventana SP142		Dako 28.8		Dako 22C3	
Cell types scored for UC	TC	IC and TC	IC		TC		TC and IC	
PD-L1 cut-offs: High/positive Low/negative	≥5% TC No visible staining	≥25% TC or IC <25% TC and IC	≥5% IC <1% IC	≥5% IC <1% IC	≥1% TC <1% TC	≥1%, ≥5% TC <1% TC	≥10% CPS NA	≥10% CPS <10% CPS
Study (Phase)	JAVELIN - UC cohort (phase 1b)	Study 1108- UC cohort (phase 1/2)	IMvigor 210 (phase 2)	IMvigor 210 (phase 2)	CM-032 UC cohort (phase 1/2)	CM-275 (phase 2)	KN-045 (phase 3)	KN-052 (phase 2)
Line of therapy	≥2L	≥1L	≥2L	1L	≥2L	≥2L	2L	1L

IC, inflammatory cells; TC, tumor cells; CPS, combined prognostic score; NA, not available; UC, urothelial carcinoma; L: line of therapy; CM, Checkmate; KN, Keynote.

## Data Availability

No new data were created or analyzed in this study. Data sharing is not applicable to this article.
